# Diameter and taper variability of gutta-percha cones adapted to TruNatomyTM and RotateTM rotary file systems

**DOI:** 10.4317/jced.59992

**Published:** 2023-01-01

**Authors:** Ignacio Martínez, Adrián Lozano, José-Luis Sanz, Leopoldo Forner, Carmen Llena

**Affiliations:** 1 DDS. Department of Stomatology, Faculty of Medicine and Dentistry, Universitat de València, 46010 Valencia, Spain; 2 MD, DDS, PhD. Department of Stomatology, Faculty of Medicine and Dentistry, Universitat de València, 46010 Valencia, Spain

## Abstract

**Background:**

Evaluate the discrepancy in diameter and taper between adapted gutta-percha cones for TruNatomyTM 26.04 (TRU04), RotateTM 25.04 (ROT04) and 25.06 (ROT06) systems and their reference files.

**Material and Methods:**

A sample of 60 gutta-percha cones and 15 rotary files was selected and divided into three groups (TRU04, ROT04, ROT06). Each group consisted of 20 cones and 5 corresponding files. They were observed under an optical microscope at x20 magnification and images of all observations were obtained. Diameters were measured with a digital ruler calibrated at 3 levels: D1, D3, D16 (mm from tip). The taper of each system of cones and files was calculated, The percentage of discrepancy between the taper of each file system and its corresponding gutta-percha cone was calculated.

**Results:**

The percentage of discrepancy between the diameter of the tested gutta-percha cones and their corresponding files varied from -7% to 21%. The smallest dimensional discrepancy between gutta-percha cone and corresponding file was found at D16 in TRU04 group. The tapers of the gutta-percha cones vs their respective files were: 2% vs 3% (TRU04), 4% vs 5% (ROT04), and 6% vs 5% (ROT06). The highest discrepancy was observed at D16 in ROT06 group. The global taper discrepancy between gutta-percha cone and its corresponding file was negative in TRU04 and ROT04 groups.

**Conclusions:**

The taper and the diameter at D1, D3, and D16 differed between all of the tested gutta-percha cones and their corresponding files. TruNatomy 26.04 files and its adapted gutta-percha cones exhibited the least discrepancy.

** Key words:**Corresponding gutta-percha, rotary file, taper, TruNatomy, Rotate, standardization.

## Introduction

The combination of a gutta-percha core with an endodontic sealer remains essential to achieve a three-dimensional filling of the root canal (1). One of the most common difficulties with the use gutta-percha is its lack of standardization (2), despite the fact that there is an international standard for its regulation (ISO 3630-1: 2019) (3).

Although manufacturing methods are being updated and new materials and technologies are being incorporated in the development of gutta-percha cones, studies continue to conclude that there is still a substantial dimensional variability between endodontic files and gutta-percha cones adapted to their dimensions, regardless of the manufacturer (4,5).

The use of gutta-percha cones with and equivalent taper and diameter to that of the last instrument used to shape the root canal is essential to achieve a correct three-dimensional apical seal (6). This requires that this instrument and its respective gutta-percha cone have been manufactured with the same standardized protocol (4).

New rotary instrumentation systems with reduced taper designs are continually appearing on the market with the aim of achieving a more conservative shaping of the root canal (7). RotateTM (VDW, Munich, Germany) and TruNatomyTM (Dentsply Sirona, Ballaigues, Switzerland) are two recently launched instrumentation systems. Associated with these systems, their respective manufacturers market gutta-percha cones with corresponding dimensions. These gutta-percha cones are not made of natural rubber latex like traditional gutta-percha cones and count with dimensions (i.e. diameter, taper) which are adapted to their respective reference file (8,9).

Given the recent introduction of these systems, to the authors’ knowledge, there are no studies that establish the concordance between the dimensions of these instruments and their specific gutta-percha cones. Accordingly, the aim of the present study was to assess the discrepancy between the diameter and the taper of the adapted gutta-percha cones of TruNatomyTM (26.04) and RotateTM (25.04 and 25.06) rotary file systems. It was proposed as a null hypothesis that no discrepancy would be found between the diameter and the taper of the files with their corresponding gutta-percha cones for each of the tested systems.

## Material and Methods

-Material selection

A sample of 60 gutta-percha cones and 15 rotary files was selected and divided into three groups: TruNatomyTM 26.04 files/cones (TRU04), RotateTM 25.04 files/cones (ROT04), and RotateTM 25.04 files/cones (ROT06). Each group consisted of 20 cones and 5 corresponding files. The characteristics of the files and the gutta-percha cones that were evaluated are presented in Table 1.

**Table 1:**
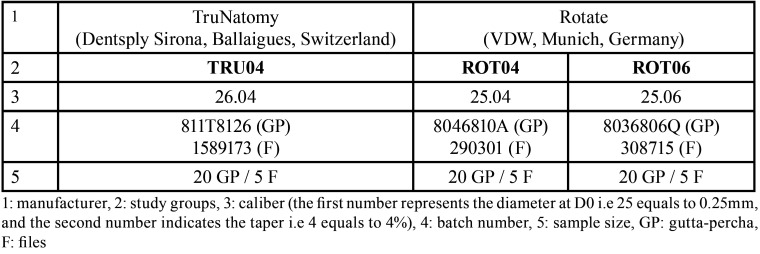
Characteristics of the selected files and gutta-percha cones.

-Optical microscope examination

Preliminarily, the selected gutta-percha points were observed under an OPMI pico optical microscope (Carl Zeiss, Jena, Germany) at 20x magnification and those that showed any irregularities and defects on its surface were discarded. 20 gutta-percha tips that were within their expiration period and that did not present any superficial microscopic defects were selected. The same process was followed for the examination of the endodontic files, resulting in the selection of 5 files from each of the assessed systems with corresponding dimensions to the selected gutta-percha cones.

**Figure 1:**
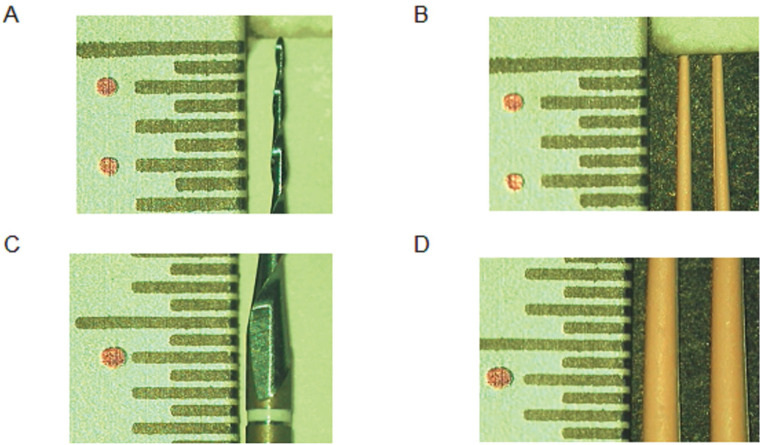
Representative images for the microscopic measurement of the diameter of the selected files and gutta-percha cones at the different reference points (D1: 1mm from the tip; D3: 3mm from the tip; D16: 16mm from the tip). The red dots in the vertical millimeter ruler mark the different reference points. Images were obtained under x20 magnification. A: D1-D3 (files), B: D1-D3 (gutta-percha), C: D16 (files), D: D16 (gutta-percha).

All samples were analyzed with the optical microscope, following standardized conditions. A flat base with two perpendicular rulers on its ends was designed, on which the gutta-percha cones and rotary files were placed for observation (Fig. 1). The vertical ruler was used to establish the following reference points: D1 (1 mm from the tip), D3 (3mm from the tip) and D16 (16mm from the tip), which were marked onto the ruler. Additionally, it served as a metric reference when capturing the microscopic image in which to posteriorly calibrate the digital ruler for the measurement of the diameters. After corroborating the proper placement of the samples and their parallelism using the microscope, an image was taken using the ArcSoft ShowBiz software (Michael Deng 1994, California, United States). Two microscopic examinations and images were taken of each gutta-percha cone and file, one for the assessment of D1 and D3 (Fig. 1A,B), and the other to assess D16 (Fig. 1C,D). All materials were observed and measured under 20x magnification.

-Diameter measurement and taper calculation

To measure the diameters at each reference point (D1, D3, and D16), a calibrated digital ruler was used with the metric reference of the microscopic images, by means of the Digital Smile Design - DSD software (Coachman and Calamita 2012, Sao Paulo, Brazil). After measuring all samples, 10% of the gutta-percha measurements were repeated and intra-observer agreement was calculated using the intraclass correlation coefficient (ICC).

**Figure 2:**
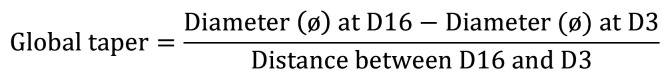
Formula.

Next, the mean diameter values in the files and gutta-percha cones and their percentage of discrepancy at D1, D3, and D16 were calculated. The taper of the cones and files was calculated using the formula for the calculation of global taper described in the ISO 6877: 2006 standard: (10), (Fig. 2).

Once the taper of the files and their corresponding gutta-percha cones had been calculated, the percentage of discrepancy between the taper of each file system and its corresponding gutta-percha cone was calculated. This value was calculated as the percentage of the difference between the taper of the gutta-percha cone and that of the file.

## Results

The intra-observer agreement was high for the three levels of measurement (between 0.81 and 0.97).

Table 2 presents the mean values of the diameters for each study group in the three reference points. In all the reference points, the diameters of the gutta-percha cones were of a larger caliber than that of their files, except for the ROT04 group at D16.

**Table 2:**
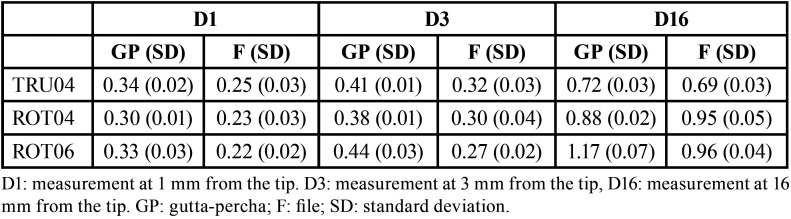
Mean diameter (mm) for each group and reference point.

At the most relevant measurement point (D1), due to the need for apical adjustment in root canal treatment, positive discrepancy values were obtained in the three systems: TRU04 (9%), ROT04 (7%), ROT06 (11%). This indicates that in the three systems, at 1mm from the tip, the diameter of the gutta-percha cone is greater than that of its corresponding file.

At D3, a positive discrepancy was also observed in each of the groups: TRU04 (9%), ROT04 (8%) and ROT06 (16%). Finally, at the most coronal reference point of the instruments (D16), positive discrepancies were obtained in TRU04 (3%), and in ROT06 (21%). However, the ROT04 file and its adapted gutta-percha exhibited a negative discrepancy of 7%, which means that the gutta-percha cones presented a lower diameter than their corresponding file at 16mm from the tip.

The file and gutta-percha cone tapers are shown in Table 3. The TRU04 and ROT04 groups presented a negative discrepancy (-1%) regarding the tapers of the gutta-percha cones with their corresponding files. On the contrary, ROT06 showed a positive discrepancy value (1%), meaning that the gutta-percha cones had a higher taper than their corresponding files.

**Table 3:**
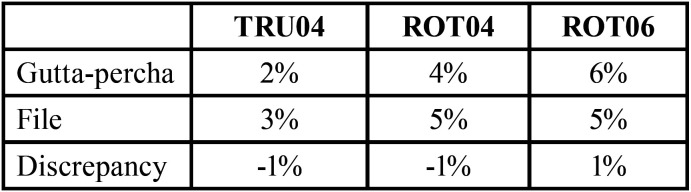
File and gutta-percha cone tapers and their discrepancy (%).

## Discussion

In the selection of gutta-percha cones during root canal treatment, dimensional variations in both diameter and taper can lead to the extrusion of gutta-percha into periapical tissues or a poor adaptation of the gutta-percha to the root canal walls (11).

The optical microscope is a validated and affordable system for the evaluation of the diameter of files and gutta-percha cones (4,11–14). Other instruments have also been used for such purpose: scanning electron microscope (5), atomic force microscope (15), calibrated ruler, (16,17) digital calibrator (16,18,19) or laser scan (20). In the present study, the combination of optical microscope visualization and the use of a calibrated ruler with marked reference points was used in order to obtain an accurate measurement of the tested materials.

The selected sample consisted of 20 gutta-percha cones and 5 rotary files per group, similar to the study of Salles *et al*. (16) and Bajaj *et al*. (17), who studied 20 gutta-percha cones and 6 rotary files per group. Other studies assessed the same number of gutta-percha cones and files (16,21). For the present study, the authors selected a higher number of gutta-percha cones than files for each system, due to the increased difficulty in achieving an optimal manufacturing of gutta-percha cones than files. Their composition and their higher deformation potential by physical and/or thermal variations hinders their standardization (2,18). This high dimensional variability of the gutta-percha cones is mainly caused by their high plasticity, which means that despite the standardization of the manufacturing process, deformations caused by the thermal changes to which they may be subjected during transport and storage can occur. (22).

In the present study, 25.04 and 25.06 caliber files were selected for RotateTM (VDW) and 26.04 for TruNatomyTM (Dentsply Sirona) rotary file systems. These files are generally the most representative of the selected rotary systems, since they present the minimum caliber to be reached during instrumentation. Additionally, they are the files that have been most frequently assessed among the studies in the field: all of the studies that compared files with their adapted gutta-percha had at least one 25 caliber file (4,16,17), except in the studies by Chesler *et al*. (5) and Mirmommahadi *et al*. (20), who used 30 and 40 caliber files, respectively.

Regarding the measurement of the diameters of the files, the most important reference point was considered to be D1 (1mm from the tip), since the adjustment of the tip of the cone is the one that allows an adequate apical seal. Points D3 and D16 were also measured to observe the dimensional variability throughout the studied sample, as well as to be able to analyze the possible discrepancy in taper between the gutta-percha cones and their corresponding files. The same reference points were used by Hartwell *et al*. (19), Gergi *et al*. (23) Chesler *et al*. (5), and Kim *et al*. (13). Other authors such as Mirmommahadi *et al*. (20) measured four apical reference points (D0, D1, D3, and D6) focusing on the concordance of the apical millimeters between files and gutta-percha cones. In the present study, two apical reference points (D1 and D3) and a coronal reference point (D16) were taken in order to be able to calculate the global taper. The use of a coronal reference point is important, since a difference between the coronal diameters of the files and the gutta-percha cones could hinder the advancement of the gutta-percha point through the root canal and its reaching towards the end of the preparation; resulting in a poor filling. Regarding the analysis of the dimensional variability of gutta-percha cones and rotary files, there are several studies that compared gutta-percha cones of various systems (15,24), rotary files alone (11-13,23), gutta-percha points and their corresponding spreaders (14,24) and, finally, rotary files with their corresponding gutta-percha cones; as in the present study (4,5,16,17,20).

To the authors’ knowledge, no other study has evaluated the concordance of the dimensions of RotateTM (VDW) and TruNatomyTM (Dentsply Sirona), and their respective gutta-percha cones. Both are multiple-file systems with continuous rotational movement. These systems were selected because their files encompass essential characteristics for root canal instrumentation, such as: high cyclical fatigue resistance, maintenance of the root canal anatomy, and preservation of a greater amount of peri-cervical dentin (25). The gutta-percha cones adapted for these systems improve their shape and adjustment in the root canal (7,9). Unlike the traditional gutta-percha cones, these cones are not manufactured with natural rubber latex and have an improvement in heat transfer, greater stability and ease of handling thanks to its grip tab (25). As a result, the assessment of the dimensional characteristics of the aforementioned files and gutta-percha cones is relevant to ensure a correct clinical performance.

To our knowledge, there are five studies that compared gutta-percha cones adapted to rotary systems, as in the present study. Salles *et al*. (16) assessed Mtwo rotary system, Bajaj *et al*. (17) Protaper Next and Wave One, and Mirmmohamadi *et al*. (20) Reciproc, Wave One, Protaper and Mtwo; all of them comparing the diameter of the gutta-percha cones with their corresponding files of the same caliber. The studies by Chesler *et al*. (5) (assessing Endosequence, K3 and Protaper), and by Haupt *et al*. (4) (assessing F360 y Reciproc) compared, in addition to the diameter, the global taper of the instruments; as in the present study. In the studies by Bajaj *et al*. (17), Mirmmohamadi *et al*. (20), and Salles *et al*. (16), all of the diameters of the gutta-percha cones were greater than those of the files, except in MTwo (40.04) (16). Haupt y cols. (4), who analyzed the diameter and taper of 20 files from two single-file systems with their corresponding gutta-percha cones, concluded that the diameter of the files in F360 was greater than that of their corresponding cones and that in Reciproc. the most coronal diameter had high percentages of difference from standard values. In all of the studies that also analyzed the taper, the global taper of the gutta-percha cones was higher than that of the files, except in the study by Chesler *et al*. (5) with EndoSequence and K3 systems.

The null hypothesis proposed in the present study was rejected. In all measurements (D1, D3, D16) of the three file / gutta-percha groups, the gutta-percha cones presented a greater diameter than their corresponding files, except in D16 of the ROT04 system, in which the diameter of the file was greater than of gutta-percha cones with a percentage of discrepancy of -7%. These data are consistent with most of the similar studies among the literature, as described above. Also, in ROT04 group, the taper of the files was greater than that of their corresponding gutta-percha cones (-1%). In the study by Salles *et al*. (16), D3 (40.04) and D1 (25.06) were the only reference points in which the file presented a greater diameter than its adapted gutta-percha cone. Mirmohammadi *et al*. (20) and Bajaj *et al*. (17), whose reference points were D1-D3-D6 and D1-D3-D11 respectively, reported that all the values of the diameters were higher in the gutta-percha cones than in their corresponding files. On the other hand, in the study by Haupt *et al*. (4), who studied Reciproc and F360 single-file systems, all the diameters of the files were found to be greater than those of the gutta-percha cones, except for D16 in R25, and D1-D16 in R50.

ROT06 group presented the highest discrepancy between the files and their corresponding gutta-percha cones. This variability was higher than 10% in the three reference points, reaching up to 21% in D16. The clinical significance of this value is given by the possibility that the gutta-percha cone cannot advance through the root canal at D16, and consequently may not reach the working length instrumented by its equivalent file.

In the present study, the lowest discrepancy was obtained for D16 reference point in TRU04 group (3%), while other studies have found the lowest discrepancies at the D1 level: Mirmohammadi *et al*. for Wave One Gold system (9,8%) (20), Bajaj *et al*. and Salles *et al*. -1% and 11%, for Wave One Gold and MTwo systems respectively (16,17).

The great variability observed in the diameter and taper between the rotary files that are used routinely and their corresponding gutta-percha cones, both in the present study and in the literature, highlights the need for clinicians to be aware of this problem and overcome it during root canal treatment. For this reason, it is still necessary to radiographically check the position and adaptation of the master cone before completing the root canal obturation, and if it does not reach the working length, select a smaller gutta-percha cone size, and calibrate the tip to the appropriate diameter. (2,26).

According to the results of the present study, it can be concluded that the taper and the diameter at D1, D3, and D16 differed between all of the tested gutta-percha cones and their corresponding files. TruNatomy 26.04 files and its adapted gutta-percha cones exhibited the least discrepancy.
